# Primary Sjögren’s syndrome-associated immune thrombocytopenia: from pathogenesis to treatment

**DOI:** 10.3389/fimmu.2026.1748373

**Published:** 2026-03-04

**Authors:** Ziqiang Zheng, Taoyuan He, Guixiang Zhang, Guohua Fu, Chang Liu

**Affiliations:** 1 Department of Rheumatology and Immunology, Central Hospital of Dalian University of Technology, Dalian, China; 2 Graduate School, Dalian Medical University, Dalian, China; 3 Department of Rheumatology and Immunology, The Second Hospital of Longyan, Longyan, China

**Keywords:** immune thrombocytopenia, platelets, predictive factors, primary Sjögren’s syndrome, treatment

## Abstract

Primary Sjögren’s syndrome (pSS) is an autoimmune disorder characterized by xerostomia and keratoconjunctivitis sicca, with approximately 10%–20% of patients developing concurrent immune thrombocytopenia (ITP). Recent studies suggest the pathogenesis of primary Sjögren’s syndrome - associated immune thrombocytopenia (pSS-ITP) may involve dysregulated TLR7 signaling, B-cell hyperactivation, and autoantibody-mediated platelet destruction. Beyond conventional therapies (e.g., glucocorticoids and intravenous immunoglobulin [IVIG]), emerging treatments have garnered increasing attention, including thrombopoietin receptor agonists (TPO-RAs), B-cell–targeted therapies, and mTOR inhibitors. Predictive models incorporating bone marrow megakaryocyte counts and autoantibody profiles may facilitate individualized treatment selection. Future multicenter clinical studies are warranted to evaluate the long-term efficacy and safety of novel agents and to explore biomarker-guided precision therapy. This review systematically summarizes the pathophysiological mechanisms of pSS- ITP, synthesizes current clinical treatment strategies, and highlights key biomarkers with potential implications for therapeutic response, aiming to provide a theoretical foundation and practical guidance for optimizing individualized therapeutic regimens.

## Introduction

Primary Sjögren’s syndrome (pSS) is a systemic inflammatory autoimmune disorder characterized by exocrine gland dysfunction, which can lead to multiorgan involvement, including hematological abnormalities (1–3). Among the clinical manifestations of pSS, hematologic disorders often serve as early indicators of the disease, with approximately 10%–20% of patients developing concurrent immune thrombocytopenia (ITP) (4–6). pSS-associated immune thrombocytopenia (pSS-ITP) is defined as an immune-mediated thrombocytopenia (platelet count <100 × 10^9^/L) secondary to pSS after excluding other underlying causes (7, 8). Clinically, ITP exhibits considerable heterogeneity, with marked variability in disease course and symptom severity across patients. It may present as a chronic, asymptomatic process or manifest as acute-onset, severe thrombocytopenia (8, 9). Prolonged pSS duration is associated with a significantly elevated risk of ITP (4). Importantly, thrombocytopenia in pSS patients is an independent risk factor for in-hospital mortality (10). Those with severe thrombocytopenia face a higher bleeding risk, increased disease activity, and poorer prognosis, all of which correlate strongly with elevated mortality rates (8, 9). The management of pSS-ITP largely mirrors that of primary ITP, relying on glucocorticoids and immunosuppressants (11). However, a subset of patients exhibits treatment resistance or relapse (12, 13). To rapidly elevate platelet counts and prevent life-threatening bleeding, high-dose glucocorticoids combined with immunosuppressants (e.g., cyclophosphamide) are frequently employed. Nevertheless, prolonged use of such regimens may lead to immunosuppression, heightened susceptibility to opportunistic infections, and drug-induced complications (e.g., hepatorenal toxicity), ultimately compromising long-term outcomes, quality of life, and imposing a substantial economic burden. Given these challenges, establishing reliable prognostic biomarkers for pSS-ITP is critical. Such markers would mitigate unnecessary interventions, tailor individualized therapy, reduce infection risks and adverse effects, and ultimately improve early remission rates and long-term prognosis. This review systematically delineates the pathophysiological mechanisms underlying pSS-ITP, synthesizes current therapeutic strategies, and evaluates key biomarkers predictive of treatment response, thereby providing a foundation for optimizing precision medicine in clinical practice.

## Pathogenesis of pSS-ITP

### Antibody-mediated mechanisms

**Figure 1:**
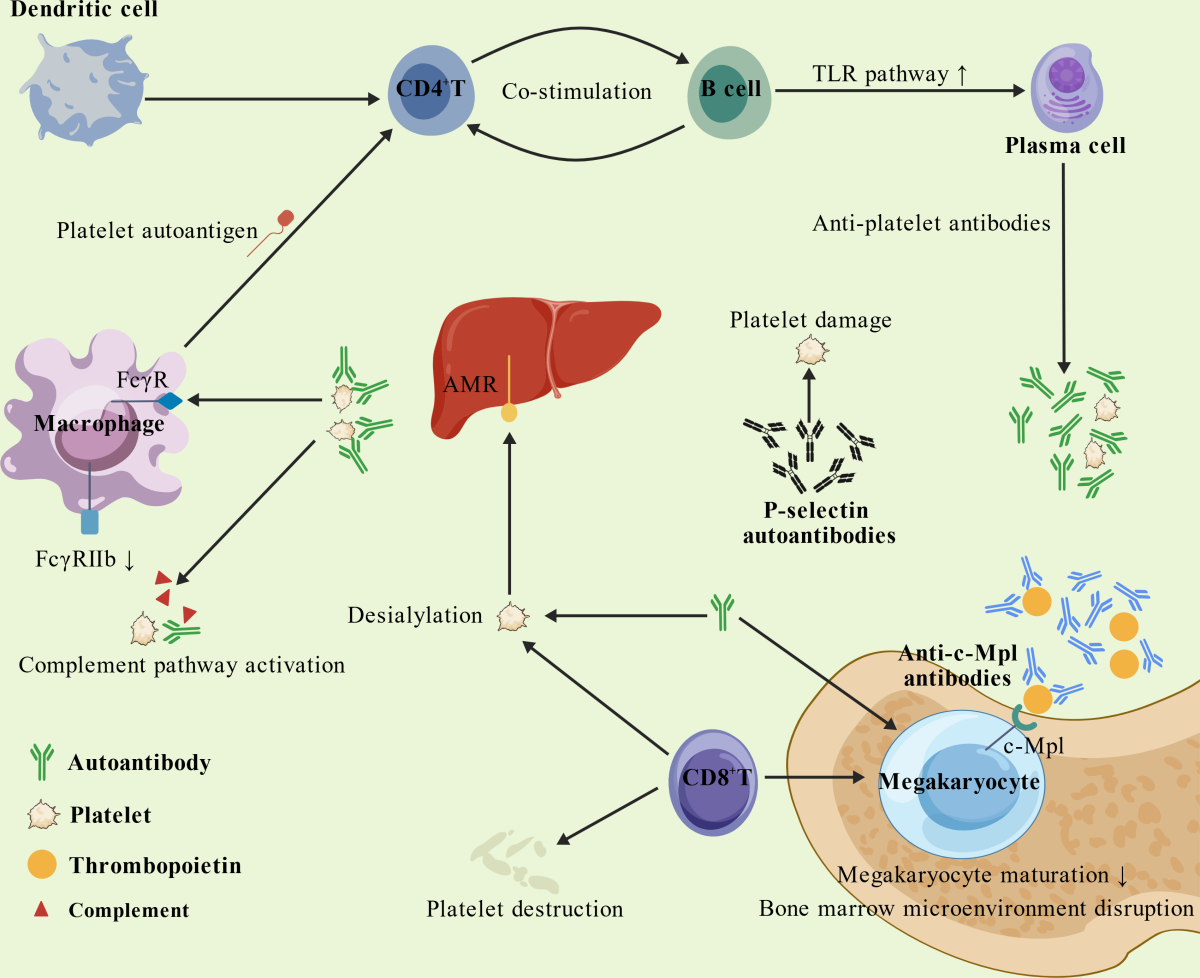
Schematic representation of the comprehensive pathogenesis of pSS-ITP. Overall, both increased platelet destruction and impaired platelet production contribute to the pathogenesis of pSS-ITP. It was created with BioGDP.com (36).

Under physiological conditions, platelet production and clearance are tightly regulated to maintain peripheral blood platelet counts within normal ranges. However, dysregulated immune responses in pSS- ITP patients disrupt this balance via accelerated platelet destruction and/or impaired platelet production, ultimately driving disease pathology (**Figure 1**). Anti-P-selectin autoantibodies may play a pivotal role. Compared to control subjects (ITP and pSS patients), pSS-ITP patients exhibit a higher positivity rate for anti-P- selectin antibodies, and antibody-positive individuals are more prone to thrombocytopenia (14). This observation suggests that anti-P-selectin antibodies not only contribute to pathogenesis but may also serve as markers of disease activity. Although antiplatelet antibodies are not diagnostic markers for pSS- ITP, their pathogenic significance is well-documented. Notably, anti-GPIb, anti-GPIIIa, and anti- GPIIb/IIIa autoantibody levels are elevated in pSS patients with thrombocytopenia compared to controls, implicating these antibodies in disease onset (15).

**Figure 2:**
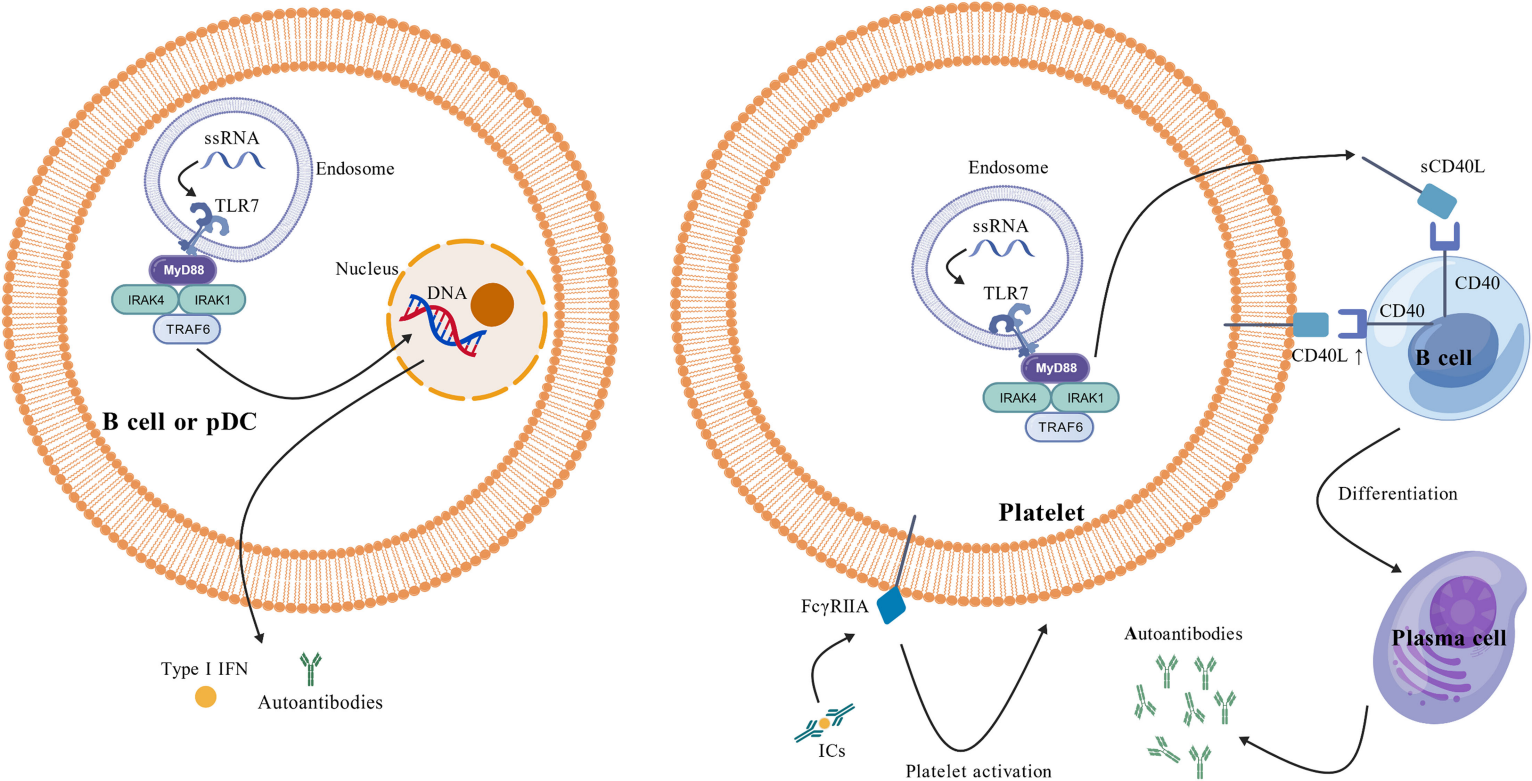
Detailed illustration of the pathogenic mechanisms underlying pSS-ITP. Toll-like receptor (TLR) signaling, particularly via TLR7, is implicated in pSS-ITP pathogenesis. In patients with pSS-ITP, aberrant recognition of ligands such as single-stranded RNA (ssRNA) by TLR7 expressed on B cells, plasmacytoid dendritic cells (pDCs), or platelets can trigger TLR7 activation and promote assembly of a complex myddosome, composed of myeloid differentiation primary response 88 (MyD88), interleukin- 1 receptor–associated kinase 4 (IRAK4) and IRAK1, and tumor necrosis factor receptor–associated factor 6 (TRAF6) (37). Myddosome formation initiates downstream signaling cascades, culminating in type I interferon (IFN) and autoantibody production by B cells and plasmacytoid dendritic cells (pDCs) and in soluble CD40 ligand (sCD40L) release from platelets. Platelet surface CD40L and released sCD40L further activate B cells, driving plasma-cell differentiation and autoantibody secretion, thereby amplifying platelet destruction. In addition, platelets express Fc gamma receptors (FcγRs), with FcγRIIA being the predominant isoform; engagement of FcγRIIA by circulating immune complexes (ICs) promotes platelet activation. pSS, primary Sjögren’s syndrome; ITP, immune thrombocytopenia. It was created with BioGDP.com (36).

During the process of autoantibody generation, the Toll-like receptor (TLR) pathway appears to play a pivotal role in the pathogenesis of Primary Sjögren’s Syndrome-associated Immune Thrombocytopenia (16). Among the various TLR subtypes, TLR7 serves as an intracellular pattern recognition receptor that is predominantly localized to the membranes of endocytic organelles, such as endosomes and lysosomes (**Figure 2**). This specific localization corresponds with its function to recognize single- stranded RNA (ssRNA) derived from intracellular viruses or endogenous sources (17). TLR7 is primarily expressed by plasmacytoid dendritic cells (pDCs) and B cells within intracellular endosomes. Activation of the TLR7 signaling pathway in these cells may lead to the production of Type I interferons (Type I IFNs) and autoantibodies (18, 19), which could subsequently induce or exacerbate autoimmune disorders, including SLE and ITP (20). Studies have revealed that the hyperactivation of the TLR7 signaling pathway in the peripheral blood of patients with PSS-ITP may facilitate the proliferation and differentiation of B cells into plasma cells, consequently leading to the production of anti-platelet antibodies (16, 21). These antibodies bind platelet surface glycoproteins, triggering complement-dependent cytotoxicity or antibody-dependent cellular phagocytosis, thereby promoting platelet hyperactivation and immune- mediated destruction (22). Mirroring human data, murine studies demonstrate that TLR7 agonist treatment induces platelet activation and thrombocytopenia in a P-selectin/P-selectin glycoprotein ligand-1 (PSGL- 1)-dependent manner (23).

It is noteworthy that platelets also express Toll-like receptors (24). Activation of the platelet TLR7 signaling pathway may result in platelet activation and the release of granules containing components such as CD40 ligand (CD40L) and P-selectin (25). Indeed, platelets serve as the primary source of soluble CD40L (sCD40L) (26), and the CD40L-CD40 signaling pathway plays a pivotal role in T-cell-mediated assistance for B-cell maturation and antibody production. In immune-mediated inflammatory disorders like Immune Thrombocytopenia, the upregulation of platelet CD40L expression appears to facilitate B- cell development and maturation. This process may ultimately drive the production of pathogenic anti- glycoprotein IIb/IIIa antibodies (27). The specific binding of these autoantibodies to glycoproteins on the platelet membrane surface could subsequently induce platelet destruction (28). Thus, anti-P-selectin and antiplatelet antibodies are inextricably linked to pSS-ITP pathogenesis.

## Bone marrow dysfunction

Bone marrow dysfunction represents another critical mechanism underlying pSS-ITP pathogenesis. Previous studies have demonstrated that anti-c-Mpl autoantibodies may contribute to pSS-ITP development by interfering with thrombopoietin (TPO) binding to its receptor c-Mpl, thereby suppressing megakaryocyte production, maturation, and subsequent platelet generation (29, 30).

Concurrently, human platelets co-express six members of the Fc gamma receptor (FcγR) family on their surface, which can be classified into activating types (e.g., FcγRI, IIa, IIIa) and inhibitory types (e.g., FcγRIIb) (25). These specific IgG receptors regulate platelet activation via signal transduction pathways mediated by Immunoreceptor Tyrosine-based Activation Motifs (ITAMs) (31). Significantly, FcγRIIa is the most abundantly expressed receptor on circulating platelets. By specifically recognizing and mediating the biological effects of circulating Immune Complexes (ICs), FcγRIIa may trigger platelet activation and autoimmune responses. Such phenomena are commonly observed in immune-mediated inflammatory disorders, including SLE and ITP (32, 33). Upon activation, platelets may undergo degranulation or release cytokines, such as sCD40L. This activity facilitates B-cell development and maturation, thereby driving the generation of anti-platelet antibodies and establishing a positive feedback loop that further exacerbates platelet destruction (27). Subsequent investigations further revealed significantly reduced expression of FcγRIIb (an inhibitory receptor) on monocytes from pSS-ITP patients, which enhances macrophage-mediated platelet phagocytosis and exacerbates platelet destruction (34, 35). Specifically, specific autoantibodies produced in patients with Sjögren’s Syndrome may also bind to platelets via similar pathways or activate FcγRs through the formation of Immune Complexes (ICs), thereby potentially exacerbating platelet clearance or destruction. Collectively, both increased platelet destruction and impaired platelet production cooperatively contribute to pSS-ITP pathogenesis.

## Current therapeutic approaches in pSS-ITP

### First-line treatments

Glucocorticoids (GC) and intravenous immunoglobulin (IVIG) remain the first-line therapies for pSS- ITP. In cases of life-threatening hemorrhage or profound thrombocytopenia, high-dose GC pulse therapy is typically required (38). This is attributed to GC’s ability to upregulate FcγRIIb receptor expression on B cells in pSS patients, thereby elevating platelet counts (39). Though GC is widely accepted for pSS-ITP treatment, the optimal long-term maintenance regimen still requires further investigation. While IVIG efficacy has been demonstrated in primary ITP, its application in pSS-ITP has also shown positive outcomes (40). However, IVIG is primarily reserved for emergency management given its short therapeutic window (2–3 weeks), high cost, and potential severe adverse effects, especially for fulminant cases requiring rapid platelet elevation (41).

## Combination strategies

Although GC monotherapy achieves an initial complete response rate of 71.6% in pSS-ITP patients, the high relapse rate (48%) frequently necessitates combination therapy with conventional immunosuppressants, among which hydroxychloroquine (HCQ) plus GC emerges as the preferred regimen in clinical practice (8). HCQ, when adopted as a second-line agent, not only demonstrates efficacy but also reduces GC dosage (42), thereby lowering risks of steroid-induced organ damage—an especially favorable profile for ITP patients with positive antinuclear antibodies (ANA). Emerging evidence highlights that combining GC with mycophenolate mofetil (MMF) significantly enhances response rates and mitigates relapse risk compared to GC alone (43), positioning this combination as a promising front- line strategy for managing pSS-ITP. Greater attention should hereafter be given to these rational drug combinations due to their synergistic immunomodulatory dynamics and tolerability profiles destined for personalized pSS-ITP therapy.

## Factors influencing treatment response in pSS-ITP

Given the incompletely elucidated pathogenesis of pSS-ITP, there is an urgent need to identify reliable clinical biomarkers for evaluating individual therapeutic responsiveness. Such markers would prevent unnecessary or ineffective interventions while reducing infection risks and drug-related toxicity, thereby facilitating early remission and improved prognosis. Recent observational studies suggest that bone marrow megakaryocyte (MK) counts may serve as a predictive biological marker, with patients exhibiting >6.5 MKs per slide demonstrating superior clinical responses to immunotherapy (44). This aligns with findings by Gan et al., where MK counts correlated with treatment failure risk in pSS-ITP (2), reflecting the critical dependence of platelet production on MK progenitor differentiation and maturation (45). As the primary site of thrombopoiesis, bone marrow cytology—already widely used in clinical practice—could emerge as a practical prognostic tool for assessing platelet productive capacity in pSS-ITP.

Beyond MK quantification, autoantibody profiles significantly influence therapeutic outcomes. Wang et al. (46) reported that anti-GPIIb/IIIa positivity was associated with higher glucocorticoid (GC) response rates, whereas anti-P-selectin antibodies predicted poorer GC responsiveness. While both antibodies are mechanistically linked to platelet destruction, their limited routine clinical detection restricts practical utility as predictive biomarkers for therapy selection. Importantly, patients carrying anti-P-selectin antibodies manifest distinct pathophysiological pathways that may necessitate alternative treatment strategies beyond conventional GC-based regimens. Emerging evidence proposed that, through the pretherapeutic autoantibody screening, such high-risk populations could benefit from early escalation to novel therapies (e.g., biologics targeting autoantibody production). Implementationally, advancing cellular assays like refined flow cytometry methodologies should be standardized for clinics to quantify pathological antibodies and guide better therapeutic choice algorithms compared with the current blanket immunosuppression paradigm in IVIG-resistant or dependent individuals, thereby inspiring precision care frameworks poised to transform outcomes.

## Clinical management challenges in refractory pSS-ITP

Refractory thrombocytopenia (RTP) is defined as failure to achieve or maintain platelet counts above 50×10^9^/L despite prednisone treatment at 1 mg/kg/day (47) or resistance to conventional immunosuppressants. While glucocorticoid (GC) combined with traditional immunosuppressants remains the standard therapy for pSS-associated ITP, approximately one-third of patients with connective tissue disease-associated ITP (CTD-ITP) show treatment failure or resistance to current standard regimens (9, 48). The management of such cases remains clinically challenging due to the lack of established guidelines and expert consensus. Current therapeutic strategies for refractory pSS-ITP primarily derive from observational studies.

Emerging evidence suggests that thrombopoietin receptor agonists (TPO-RAs), as non- immunosuppressive agents, may offer new therapeutic potential. Recent case reports indicate that eltrombopag—a TPO-RA—demonstrates both efficacy and safety in treating refractory pSS-ITP without significant adverse effects (12). Another notable alternative is sirolimus (mTOR inhibitor). Preliminary studies in CTD-ITP have shown promising results, with a 75% overall response rate observed in refractory pSS-ITP patients receiving sirolimus and no adverse events reported during 6-month follow- up (49). Critically, these findings require validation through larger randomized controlled trials due to the current limited sample sizes.

Rituximab has emerged as another viable option for refractory pSS-ITP. Earlier studies established the efficacy and safety of low-dose rituximab in this population (50). Subsequent research by Jiang et al. extended these findings, demonstrating an 80.95% overall response rate with good tolerability in refractory CTD-ITP patients (including pSS and SLE) (51). More recently, Sun et al. reported superior sustained remission rates with rituximab compared to cyclosporine A (81.8% *vs* 53.5% at 6 months) in CTD-ITP patients (13). Collectively, available evidence positions rituximab as a promising therapeutic alternative for refractory pSS-ITP.

## Emerging targeted therapies for pSS-ITP

B cell surface receptors, particularly Toll-like receptors (TLRs) 7 and 8, play pivotal roles in immune- mediated inflammatory diseases. Genetic and *in vivo* evidence confirms that abnormal recognition of RNA-containing autoantigens by TLR7/8 triggers autoimmune pathogenesis (52). In pSS-ITP patients, excessive TLR7 pathway activation promotes B-cell proliferation, plasma cell differentiation, and subsequent antiplatelet antibody production, ultimately accelerating peripheral platelet destruction (16). Notably, Hawtin et al. demonstrated that MHV370 effectively suppresses TLR7/8-mediated B-cell activation both *in vitro* (eliminating downstream B cells) and *in vivo* (preventive/therapeutic administration blocking cytokine secretion) (52). Currently under phase 2 trials for Sjögren’s syndrome and mixed connective tissue disease (53), this TLR-targeted strategy may reduce pathological antibody production in pSS-ITP—though randomized controlled trials remain essential to validate efficacy and safety.

Furthermore, the CD40L-CD40 axis critically governs T-cell-dependent B-cell responses. In immune- mediated inflammatory diseases (IMIDs) like ITP, elevated platelet CD40L expression drives pathogenic anti-GPIIb/IIIa antibody generation (27). While CD40/CD40L blockade shows therapeutic potential for autoimmune diseases (including pSS) (54), safety concerns persist. As observed in clinical trials of ruplizumab (anti-CD40L monoclonal antibody) for chronic refractory primary ITP, increased thrombotic events substantially limited its applicability (55). Consequently, future studies must thoroughly evaluate risk-benefit profiles before considering CD40L-directed therapies for pSS-ITP.

## Future perspectives on pSS-ITP management

While the pathogenesis of pSS-ITP remains incompletely understood, establishing reliable clinical parameters to predict treatment response is critically needed. Currently, the therapeutic landscape for pSS-ITP is limited by a striking lack of randomized controlled trials and high-quality studies investigating novel targets, significantly constraining treatment optimization. Immunosuppressants remain the mainstay treatment despite their adverse effects, including increased infection risk and organ damage that negatively impact patient prognosis and quality of life. Over the past decade, non- immunosuppressive agents like TPO-RAs have shown efficacy in primary ITP, with emerging retrospective studies and case reports suggesting similar promise for pSS-ITP, particularly in refractory cases failing conventional therapy. However, multiple crucial questions remain unanswered regarding optimal treatment timing, selection criteria, management of refractory disease, and prevention of overtreatment. Though observational studies have identified potential therapies for refractory pSS-ITP, high-quality clinical trials validating these approaches are lacking. Future research priorities should focus on developing stratification models to identify patients most likely to benefit from specific treatments, as well as investigating platelet-targeted therapies based on emerging evidence of platelets’ role in immune-mediated inflammatory diseases. The mechanistic insights from SLE-ITP treatment strategies targeting platelet activation and platelet-immune cell interactions may provide valuable directions for pSS-ITP research (56). Addressing these knowledge gaps through multicenter collaborations will be essential to advance personalized treatment paradigms for this challenging condition.
