# Correction: Primary Sjögren’s syndrome-associated immune thrombocytopenia: from pathogenesis to treatment

**DOI:** 10.3389/fimmu.2026.1827822

**Published:** 2026-03-24

**Authors:** Ziqiang Zheng, Taoyuan He, Guixiang Zhang, Guohua Fu, Chang Liu

**Affiliations:** 1 Department of Rheumatology and Immunology, Central Hospital of Dalian University of Technology, Dalian, China; 2 Graduate School, Dalian Medical University, Dalian, China; 3 Department of Rheumatology and Immunology, The Second Hospital of Longyan, Longyan, China

**Keywords:** immune thrombocytopenia, platelets, predictive factors, primary Sjögren’s syndrome, treatment

The order of authors in the author list of the published paper was erroneously given as:

“Chang Liu, Ziqiang Zheng, Taoyuan He, Guixiang Zhang, Guohua Fu”.

The correct author list reads:

“Ziqiang Zheng, Taoyuan He, Guixiang Zhang, Guohua Fu, Chang Liu”.

The original version of this article has been updated.

